# Highly Sensitive Detection of Melamine Based on the Fluorescence Resonance Energy Transfer between Conjugated Polymer Nanoparticles and Gold Nanoparticles

**DOI:** 10.3390/polym10080873

**Published:** 2018-08-06

**Authors:** Cui-jiao Zhang, Zhi-yan Gao, Qiu-bo Wang, Xian Zhang, Jin-shui Yao, Cong-de Qiao, Qin-ze Liu

**Affiliations:** 1 School of Materials Science and Engineering, Qilu University of Technology (Shandong Academy of Sciences), Jinan 250353, China; 13256729106@163.com (C.Z.); zhiyangao98@126.com (Z.G.); wangqbqlu18@163.com (Q.W.); yaojsh@qlu.edu.cn (J.Y.); cdqiao@qlu.edu.cn (C.Q.); liuqinze01@163.com (Q.L.); 2 Shandong Provincial Key Laboratory of Processing and Testing Technology of Glass and Functional Ceramics, Key Laboratory of Amorphous and Polycrystalline Materials, Qilu University of Technology, Jinan 250353, China

**Keywords:** fluorescent assay, fluorescence resonance energy transfer, conjugated polymer nanoparticles, gold nanoparticles, melamine

## Abstract

Adding melamine as additives in food products will lead to many diseases and even death. However, the present techniques of melamine detection require time-consuming steps, complicated procedures and expensive analytical apparatus. The fluorescent assay method was facile and highly sensitive. In this work, a fluorescence resonance energy transfer (FRET) system for melamine detection was constructed based on conjugated polymer nanoparticles (CPNs) and gold nanoparticles (AuNPs). The energy transfer efficiency is up to 82.1%, and the system is highly selective and sensitive to melamine detection with a lower detection limit of 1.7 nmol/L. Moreover, the interaction mechanism was explored. The results showed that the fluorescence of CPNs were firstly quenched by AuNPs, and then restored after adding melamine because of reducing FRET between CPNs and AuNPs. Lastly, the proposed method was carried out for melamine detection in powdered infant formula with satisfactory results.

## 1. Introduction

As we all know, melamine (C_3_H_6_N_6_), an odorless and white heterocycle compound, is widely used in the manufacturing of plastics, laminates, dyes, fabrics, and fertilizers [1,2]. Moreover, it is often added as an additive in food products to increase the amount of protein detection due to its high nitrogen concentration (66% by mass) [3]. However, this will result in kidney stones, urinary system damage and even death [4]. Melamine is ingested beyond the safety limit of 8 μmol/L for infant formula in China and 20 μmol/L in the USA and EU [5]. Therefore, it is quite necessary to develop a cheap, sensitive and reliable method for melamine detection.

Nowadays, many analytical approaches of melamine detection, such as mass spectroscopy (MS) [6], potentiometric [7], gas chromatography mass spectrometry (GC-MS) [8] and electrochemical [9] have been applied. But these methods require expensive and complicated analytical instruments, are time-consuming and require experienced operators, which limit their wide applications in routine analysis.

Fluorescence assays based on FRET have attracted much interest due to significant merits including high sensitivity, simple instruments, operational simplicity and test rapidity, which can be observed when the space distance between the acceptor and the donor is very close (7~10 nm). Many quantum dots (QDs) and organic dyes were recognized as efficient FRET-based melamine donors such as CdTe [10,11], fluorescein [12], rhodamine B [3,13] and so on. However, these materials suffer from high toxicity, poor stability and photobleaching, which lead to irreproducible fluorescence signals for further analysis. Recently, several methods have been reported based on gold nanoparticles (AuNPs) by color changes of the solution to detect melamine [14,15]. Melamine can reduce the stability of AuNPs and result in their aggregation. Meanwhile, the color of AuNPs solution turned to deep blue from wine red. This variation can be lightly observed by the naked eye and UV-Vis absorption measurement [16,17]. Therefore, AuNPs-based colorimetric detection melamine method is rapid and simple [18]. However, interference substances in milk, which compete with melamine during AuNPs binding, often result in a false positive response. To solve this problem, AuNPs have been successfully utilized as the acceptor for the FRET system owing to high extinction coefficients, conductivity, excellent biocompatibility and size-dependent optical properties [19,20]. In addition, CPNs have been developed as fluorescence probes for tumor imaging [21,22], neuroinflammation [23,24] and cell tracking [25]. Compared to organic dyes and traditional QDs, the superiorities of CPNs include easy preparation, extraordinary fluorescence brightness, low toxicity and good biocompatibility [26,27]. For all we know, the melamine detection based on FRET of CPNs and AuNPs has not been reported.

**Scheme 1:**
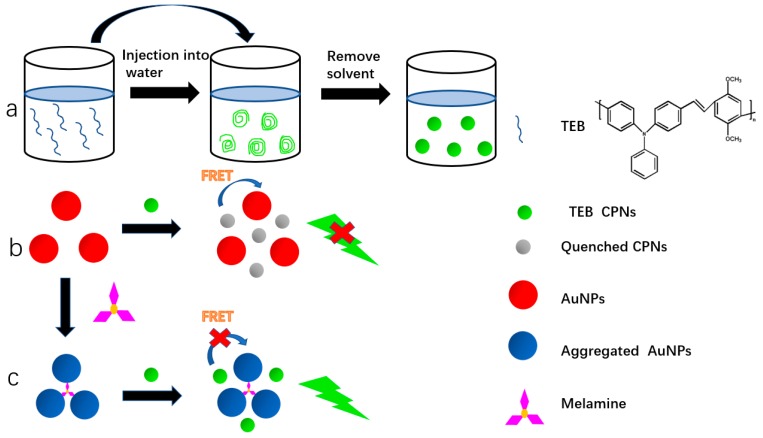
The mechanism diagram of melamine detection by FRET based on CPNs-AuNPs; (**a**) the preparation process of CPNs; (**b**) the fluorescence of CPNs was quenched by the FRET of CPNs-AuNPs; (**c**) the fluorescence of CPNs was restored after adding melamine.

Herein, an efficient “turn-on” fluorescence sensor for melamine detection was designed based on melamine-induced reduction of FRET efficiency between AuNPs and CPNs. The key diagram of our method is displayed in Scheme 1. The fluorescence of CPNs was well quenched when the CPNs nanoparticles were apt to close the surface of AuNPs. However, the aggregation of AuNPs via N-Au interaction after adding melamine would reduce the FRET of CPNs-AuNPs and promote the fluorescence recovery of CPNs. This is because the spectral overlap between the absorption spectrum of AuNPs and the fluorescence emission spectrum of CPNs will alter after adding melamine, and decrease the energy transfer efficiency of CPNs-AuNPs. Furthermore, the above technique has been well used for melamine detection in real milk powder samples.

## 2. Experimental

### 2.1. Chemicals and Apparatus

HAuCl_4_·4H_2_O and sodium citrate were gained from Shanghai Chemical Reagent Co. (Shanghai, China). Melamine was obtained from Aladdin Reagent Company (Shanghai, China). l-cysteine, l-histidine, Vitamin C, Glucose, NaOH, Ca(NO_3_)_2_·4H_2_O, FeCl_3_·6H_2_O, KNO_3_, Na_2_SO_4_, MgCl_2_, NaCl were purchased from Tianjin Chemical Works (Tianjin, China). All chemical reagents were used without further purification. The milk powder was obtained from the local supermarket.

Absorption spectra were measured on UV-2500 spectrophotometer (Shimadzu, Shuzhou, China). Fluorescence measurements were finished with F-7000 FL spectrometer (Hitachi, Tokyo, Japan). Transmission electron microscopy (TEM) were given on a JEM–2100 transmission electron microscope (JEOL Ltd., Musashino, Japan). The zeta potentials and size distribution were estimated by dynamic light scattering (DLS) on a Zetasizer Nano ZS90 (Malvern, Worcestershire, UK).

### 2.2. Preparation of AuNPs

AuNPs were synthetized via the citrate reduction method described in previous reports [28,29]. Typically, 1 mL aqueous solution of HAuCl_4_ (1%) and 100 mL ultrapure water were firstly heated to boiling, and sodium citrate solution (1%, 4 mL) was added quickly. The reaction was further refluxed for 20 min, and then stored at 4 °C. The sizes of AuNPs were measured to be about 24 nm by DLS (Figure S1a) and TEM (Figure 4A). The molar concentration of AuNPs was calculated to be approximately 2.96 × 10^−9^ mol L^−1^ by Lambert-Beer law using the molar extinction coefficient of reported AuNPs in the literature (2.7 × 10^8^ mol^−1^ L cm^−1^ at 520 nm) [16].

### 2.3. Preparation of CPNs

The Conjugated polymer TEB (Scheme 1) was synthesized according to our previous method [30]. The *M*_w_ and *M*_w_/*M*_n_ of TEB was 7032 and 1.174, respectively. The nanoparticles of TEB were carried out using the procedure described in the literature [31,32]. As shown in Scheme 1a, TEB were fully dissolved in 10 mL THF to prepare a 20 μg/mL stock solution, and then the solution was rapidly injected into 20 mL of ultrapure water in a sonication bath. After 15 min, THF was removed by rotary evaporation and the solution was concentrated to 10 mL, and then filtered through a 0.22 μm micro-filter. The sizes of CPNs were measured to be about 50.7 nm by DLS (Figure S1c).

### 2.4. Fluorescence Analysis

Typically, at room temperature, 100 μL CPNs (20 μg/mL) was mixed with as-prepared original AuNPs with different concentrations and incubated for 10 min. Then the emission spectra of solutions were measured.

The AuNPs solution was diluted with ultrapure water to 2.37 × 10^−9^ mol L^−1^ for further use. The as-prepared AuNPs solution was mixed with different concentrations (0–2 μmol/L) of melamine solution, and the mixture liquid was incubated for 15 min at room temperature. Then, 100 μL CPNs was injected into the above liquid and diluted to 4 μg/mL with PBS (pH = 7). Finally, the above mixture was in a still state for 10 min before the spectral measurement.

### 2.5. Pretreatment of Real Samples

Milk powder was firstly processed according to the previous reports [11,33]. Briefly, 2 g of milk powder was fully dissolved in 5 mL of acetonitrile and 15 mL of 1% (*w*/*v*) trichloroacetic acid. Then the mixed liquid was ultrasonically treated and centrifuged at 10,000 rpm for 15 min. Lastly, the liquid was filtered by a 0.22 μm filter, and further diluted 25-fold to acquire the testing samples.

## 3. Results and Discussion

### 3.1. Optical Characteristics of AuNPs and CPNs

**Figure 1:**
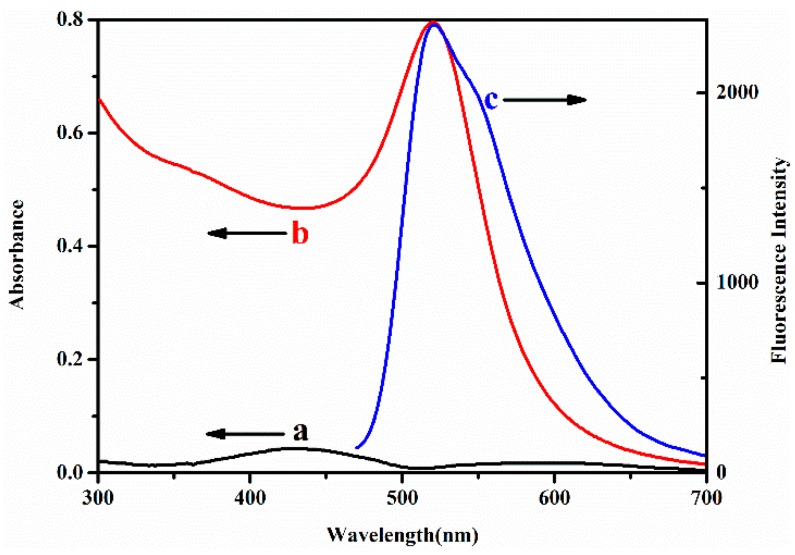
The absorption spectra of the CPNs (a) and AuNPs (b) and fluorescence emission spectrum of CPNs (c).

The AuNPs were prepared by the reported method [34]. The as-prepared AuNPs was negatively charged with a zeta potential of 8.43 mV (Figure S2a). The absorption and fluorescence emission spectra of AuNPs and CPNs were measured and shown in Figure 1. A broad overlap from 480 to 600 nm between them was found, which implied that the FRET may occur between CPNs (donor) and AuNPs (acceptor). Consequently, the fluorescence intensity of CPNs may be evidently quenched or reduced if CPNs and AuNPs coexist.

**Figure 2:**
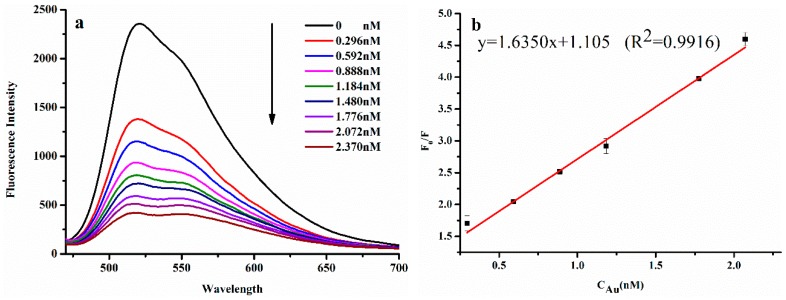
(**a**) Fluorescence emission spectra of CPNs with the increased concentrations of AuNPs; (**b**) the linear fitting plot of *F*_0_*/F* values at 520 nm versus AuNPs concentrations from 0 to 2.370 nM, CPNs: 0.187 μM.

To identify the above hypothesis, the fluorescence intensities of CPNs were investigated with the increased concentrations of AuNPs (Figure 2). The energy transfer efficiency (*E*) was measured and gained by the following equation [34]:

(1) $$\;E=1-I/I_{0}\;$$

where *I* and *I*_0_ are the fluorescence intensity of the donor (CPNs) after and before the addition of acceptor (AuNPs), respectively. *E* obtained the value of 82.1% according to Equation (1). The fluorescence intensities of CPNs were contravariant to the concentrations of AuNPs. The fluorescence quenching efficiency of CPNs by AuNPs was measured by the Stern–Volmer equation [35]:

(2) $$\;\frac{F_{0}}{F}=K_{SV}\times C_{Q}+1\;$$

where *F*_0_ and *F* denoted the fluorescence intensity of CPNs before and after the addition of AuNPs, respectively. *C_Q_* is the concentration of AuNPs. The curve of *F*_0_*/F* to *C_Q_* was linearly fitted and shown in Figure 2b, and the Stern–Volmer equation is described below:

(3) $$\frac{F_{0}}{F}=1.635\;C_{Q}+1\;\;\left(R^{2}\;=\;0.9916\right)\;$$

*K*_SV_ was the quenching constant and gained to be 1.635 × 10^9^ (mol^−1^ L)^−1^. This analytical parameter is better than those in previous reports [36,37].

### 3.2. Interaction of AuNPs-CPNs with Melamine

**Figure 3:**
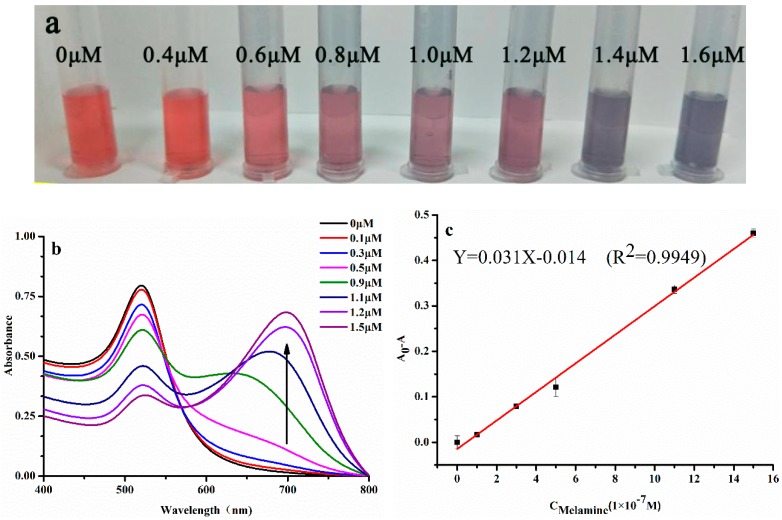
(**a**) The visual color change of the AuNPs with different concentrations of melamine; they were marked on the tubes; (**b**) absorption spectra of CPNs-AuNPs with different concentrations of melamine. AuNPs: 2.37 nM; (**c**) a linear relationship (at 522 nm) between A_0_–A and melamine concentrations; A_0_ and A are the absorbance in the absence and presence of melamine, respectively.

The color variation and absorption spectra of AuNPs solutions with the increased concentrations of melamine are shown in Figure 3a,b. The obvious color variations from red to deep blue were found in the concentrations of melamine from 0.4 to 1.6 μM. The characteristic absorption of AuNPs gradually decreased at 522 nm and showed slightly red-shifted. Meanwhile, a longer wavelength absorption band (700 nm) appeared gradually. The possible reason may be that melamine could strongly coordinate to AuNPs by the amine groups, and finally lead to the aggregation and destabilization of dispersed AuNPs [38,39,40]. Meanwhile, the red-shifted absorption of Au NPs declined the spectral overlap between the emission spectrum from CPNs and the absorption spectrum of AuNPs and reduced *E*.

A good linear relationship between the decreased absorbance at 522 nm and the concentrations of melamine was gained from 0.1–1.5 μmol/L (*R*^2^ = 0.9949, Figure 3c). The relative standard deviation was 0.8% for seven repeated measurements, which illustrated the reliability of the proposed method. The limit of detection *(LOD* = 3δ/*K*, where *K* represents the slope and δ is the standard deviation) was measured to be 6.8 nmol/L. This analytical parameter is better than those in previous reports (Table 1).

**Figure 4:**
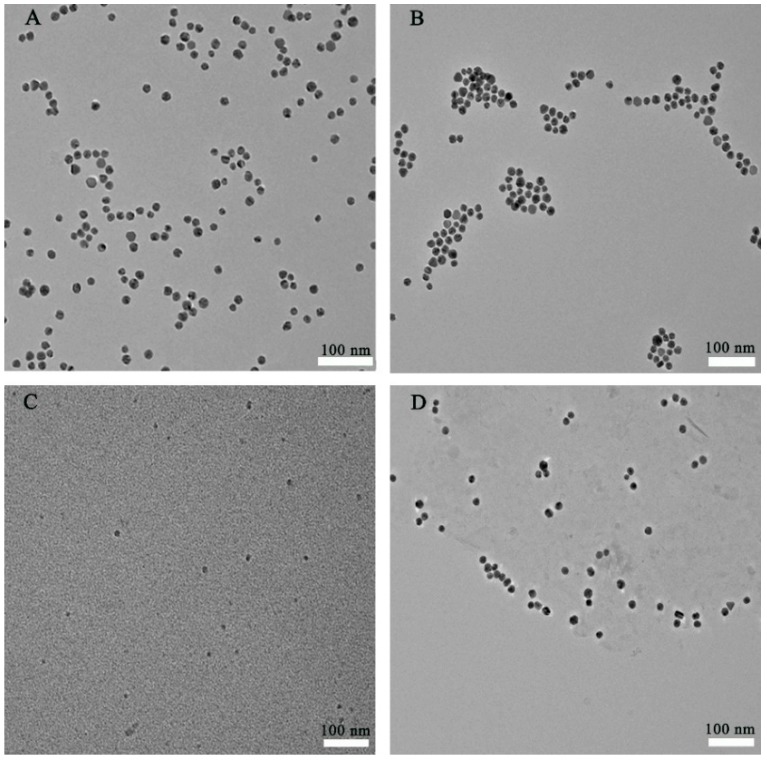
(**A**) TEM images of the original AuNPs; (**B**) TEM images of the AuNPs in the presence of melamine; (**C**) TEM images of the CPNs; (**D**) TEM images of the AuNPs in the presence of CPNs.

TEM observations could further confirm the aggregation of dispersed AuNPs owing to the addition of melamine. From Figure 4A, the original AuNPs were uniform in size and spherical in shape. After mixing with CPNs, AuNPs were still in a scattered state, indicating that the addition of CPNs did not affect the dispersity of AuNPs (Figure 4D). However, obvious aggregation of AuNPs could be found after adding melamine (Figure 4B). The DLS data also indicated the same conclusion because the average sizes of AuNPs were 24 and 58.8 nm in the absence and presence of melamine (Figure S1a,b), respectively. The results were fully consistent with the spectral variation in Figure 3. Hence, the fluorescence intensity of CPNs could be monitored in the AuNPs-CPNs system by FRET, which afforded a feasible and new strategy for melamine detection. Actually, the addition of melamine led to the aggregation of AuNPs accompanying color change, which have been used for the visual sensing for melamine detection in infants and liquid milk [18]. However, these colorimetric techniques usually displayed lower sensitivity to melamine in comparison with fluorescence analytical methods. It is greatly hopeful to develop a simple way based on the FRET method with the higher sensitivity and the lower detection limit, and fluorescence signals can replace the variation of absorption.

### 3.3. FRET-Based AuNPs-CPNs Emission Response to Melamine

**Figure 5:**
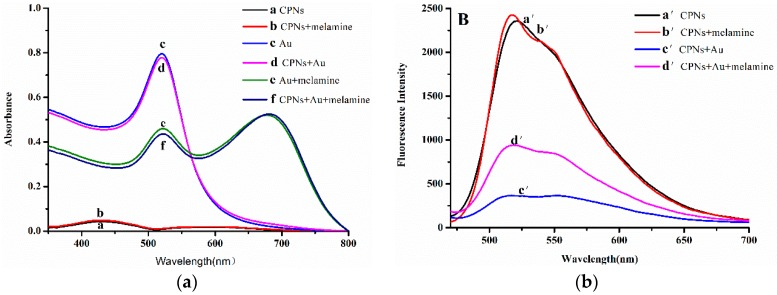
(**a**) Absorption spectra; a: CPNs; b: CPNs and melamine; c: AuNPs; d: AuNPs and CPNs; e: AuNPs and melamine; f: mixture of CPNs, AuNPs and melamine; (**b**) fluorescence spectra; a’: CPNs; b’: CPNs and melamine; c’: AuNPs and CPNs; d’: mixture of CPNs, AuNPs and melamine; CPNs: 0.187 μM; AuNPs: 2.37 nM; melamine: 0.8 μM.

The FRET system of CPNs-AuNPs would be modulated by the concentrations of melamine, and the fluorescence intensity of CPNs would change accordingly because melamine congregated the AuNPs (Scheme 1). The relationship between melamine and the FRET system was further investigated to validate the competition effect between CPNs and melamine. As displayed in Figure 5, it was proved that CPNs and melamine did not directly interact because the absorption and fluorescence intensity of CPNs (curves a and a’ in Figure 5) were identical to the mixture of CPNs and melamine (curves b and b’ in Figure 5). The characteristic absorption of AuNPs displayed no distinct variation before and after adding CPNs (curve c and d in Figure 5a), indicating that CPNs and AuNPs were not directly interacting. In addition, the characteristic absorption of AuNPs-melamine were identical in the absence or presence of CPNs (curves e and f in Figure 5a), which indicated that the aggregation of AuNPs was the result of the addition of melamine. The same conclusion could be got by no change for the average size of AuNPs-melamine before and after adding CPNs (Figure S1b,d). The fluorescence intensity of CPNs-AuNPs was evidently quenched owing to the FRET (curve c’ in Figure 5b) when CPNs and AuNPs were mixed together. Nevertheless, the addition of melamine could distinctly recover the fluorescence of CPNs curve d’ in Figure 5b. In addition, the fluorescence quantum yields (*Ф*s) of CPNs in the absence and presence of AuNPs and melamine have been calculated using a solution containing coumarin 307 (*Ф* = 0.56, in methanol) standard as the reference according to the equation reported [42]. The values of *Ф*s were listed in Table 2. The change of *Ф*s was similar to that of fluorescence. With the addition of AuNPs, *Ф*s of CPNs reduced from 0.22 to 0.0050. However, further adding melamine, *Ф*s of CPNs were restored. But no obvious variation was found for *Ф*s of CPN in the presence of melamine.

**Figure 6:**
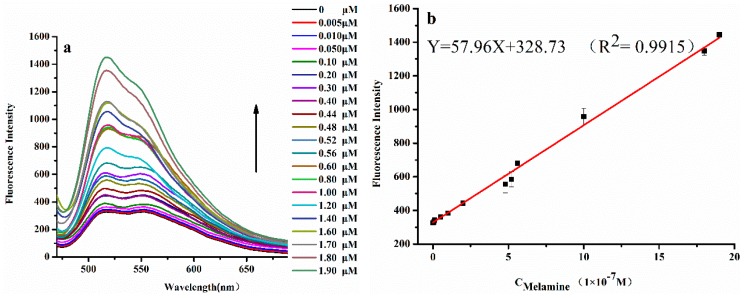
(**a**) Fluorescence emission spectra of CPNs-AuNPs nanosensor with different concentrations of melamine from 0.05 to 19 × 10^−7^ mol/L. CPNs: 0.187 μM. AuNPs: 2.37 nM; (**b**) a linear relationship with respect to the enhanced fluorescence intensities and melamine concentrations.

The present technique for melamine detection was further researched under the optimal conditions. From Figure 6a, the fluorescence emission intensities of AuNPs-CPNs were recovered gradually with the increased concentrations of melamine. The increased fluorescence intensity and melamine concentrations had a fine linear relationship from 5 nmol/L to 1.9 μmol/L (*R*^2^ = 0.9915, Figure 6b). The detection limit was calculated to be 1.7 nmol/L. This analytical parameter is very low in comparison with the reported literatures (Table 1). The relative standard deviation by operating 10 repeated measurements of melamine (1.9 μmol/L) was 1.2%, which illustrated that the fluorescence response of AuNPs-CPNs toward melamine was highly reproducible.

### 3.4. Selective Studies and the Application to Real Samples

**Figure 7:**
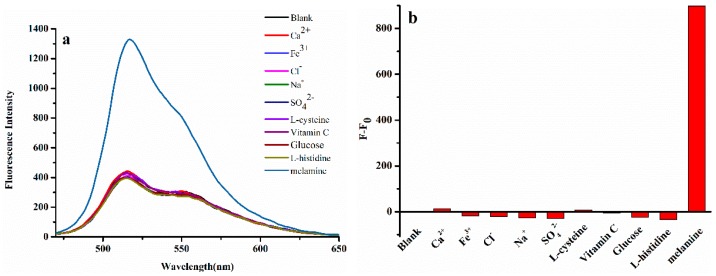
(**a**) Selectivity of the CPNs-AuNPs toward melamine; (**b**) the Bar graph of CPNs-AuNPs selectivity. The concentrations of interferences and melamine were both 2 μM.

To evaluate the method used in the specific detection of melamine, the potential interfering substances in real sample and melamine (2 μmol/L), such as Ca^2+^, Fe^3+^, Na^+^, SO_4_^2−^, Cl^−^, vitamin C, cysteine, histidine and glucose, were examined, respectively. From Figure 7a,b, no obvious interferences were found, which indicated that the AuNPs-CPNs towards melamine detection is highly selective.

To further prove the present method, the amount of melamine was directly spiked into milk powder after sample pretreatment, and the melamine-doped milk samples were further handled by the procedure described in Section 2.4. The analytical results are given in Table 3. The recovered values of the three milk powder samples varied from 95.93% to 100.8%. These analytical parameters are better than those in previous reports (Table 1), which indicated that the proposed method was suitable for melamine detection in real samples.

## 4. Conclusions

A new and effective FRET based on the system of CPNs-AuNPs was developed for melamine detection. In the presence of melamine, the FRET between AuNPs and CPNs was gradually inhibited, resulting in the fluorescence recovery of CPNs. Melamine could be detected with a lower detection limit of 1.7 nmol/L under the optimum experimental conditions. Moreover, the proposed technique was used for the detection of melamine in real samples with the satisfactory experimental results.

## Figures and Tables

**Table 1:** Comparison of different methods for the determination of melamine.

Method	LOD (μM)	Ref.	Recovery (%)
HPLC	0.79	[41]	97.2–101.2
Turn-off fluorescence CdTe quantum dots	0.31	[11]	103–104
CdTe QDs and Rhodamine B	0.01	[35]	99.2–104
FRET/carbon dots and Au nanoparticles	0.036	[5]	90.5–111.4
FRET between CdTe/CdS QDs and AuNPs	0.03	[2]	90.0–101
Au nanoparticles and Melamine	0.0068	This work (1)	-
FRET/CPNs and Au nanoparticles	0.0017	This work (2)	95.93–100.8

**Table 2:** The fluorescence quantum yields of CPNs under difference conditions.

Components	Concentration (nΜ) (CPNs/AuNPs: /Melamine)	*Ф*
CPNs	187/0/0	0.22
CPNs +Au NPs (1)	187/1.184/0	0.022
CPNs +Au NPs (2)	187/2.370/0	0.0050
CPNs + AuNPs + melamine (1)	187/2.370/300	0.0086
CPNs + AuNPs + melamine (2)	187/2370/1200	0.016
CPNs + melamine	187/0/1200	0.22

*Ф*: Fluorescence quantum yield.

**Table 3:** The application of the proposed method for melamine detection in milk samples spiked with different concentrations of melamine (*n* = 3, average value of three determinations).

Sample	Amount Added (10^−7^ M)	Amount Founded (10^−7^ M)	Recovery (%)	R.S.D. (%)
1	5.2	5.080	97.74	5.0
2	14	13.430	95.93	4.1
3	18	18.144	100.8	2.7

R.S.D.: relative standard deviation.

## Supplementary Materials

The following are available online at http://www.mdpi.com/2073-4360/10/8/873/s1.
