# Supplementary material for: Highly Sensitive Detection of Melamine Based on the Fluorescence Resonance Energy Transfer between Conjugated Polymer Nanoparticles and Gold Nanoparticles
Source: Polymers (Basel). 2018 Aug 6;10(8):873. doi: 10.3390/polym10080873 (PMC6403951; doi:10.3390/polym10080873)
Supplement: Supplementary file 1 [file polymers-10-00873-s001.pdf]

Supporting information

Highly Sensitive Detection of melamine based on the fluorescence resonance energy transfer between conjugated polymer nanoparticles and gold nanoparticles

Cui-jiao Zhang [a, b], Zhi-yan Gao [a, b], Qiu-bo Wang [a, b], Xian Zhang* [a, b], Jin-shui Yao [a, b], Qin-ze Liu [a, b], Cong-de Qiao [a, b]

[a] School of Materials Science and Engineering，Qilu University of Technology (Shandong Academy of Sciences)，Jinan 250353，China

[b] Shandong Provincial Key Laboratory of Processing and Testing Technology of Glass and Functional Ceramics; Key Laboratory of Amorphous and Polycrystalline Materials; Qilu University of Technology, Jinan 250353, China

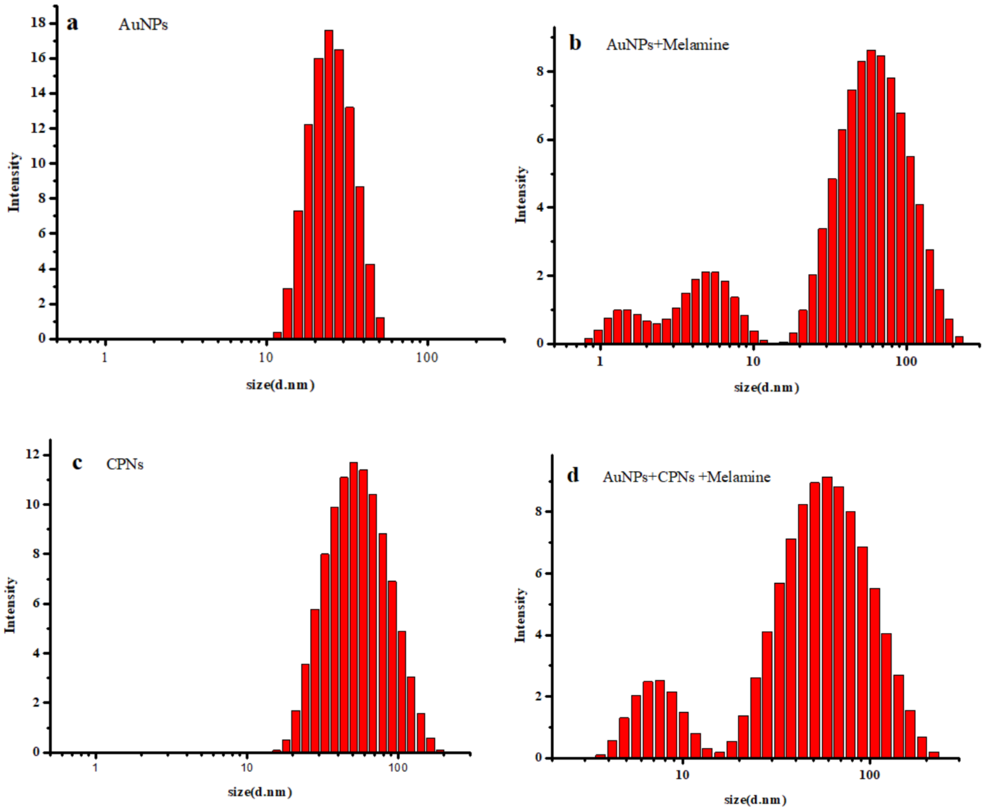

Fig. S1. (a) Size distribution of AuNPs (average size = 24.4 nm); (b) size distribution of AuNPs with the addition of melamine (average size = 58.8 nm); (c) size distribution of CPNs (average size = 50.7 nm); (d)

size distribution of AuNPs and CPNs with the addition of melamine (average size = 58.8 nm). Concentration of AuNPs:2.37 nM; concentration of melamine: 100 mol L^{-1}; concentration of CPNs: 10 μg/mL.

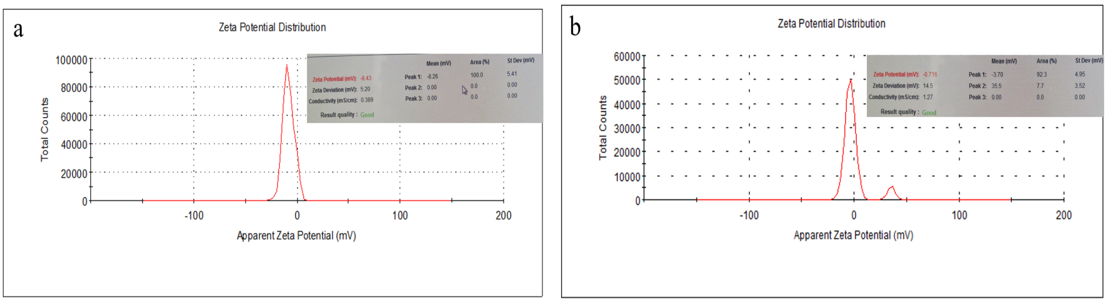

Fig. S2. Zeta potential of AuNPs (a) and CNPs (b) at pH = 7.0, respectively.
